# Patient-identified information and communication needs in the context of major trauma

**DOI:** 10.1186/s12913-018-2971-7

**Published:** 2018-03-07

**Authors:** Sandra Braaf, Shanthi Ameratunga, Andrew Nunn, Nicola Christie, Warwick Teague, Rodney Judson, Belinda J. Gabbe

**Affiliations:** 1 Department of Epidemiology and Preventive Medicine, Monash University, The Alfred Centre, Commercial Rd, Melbourne, VIC 3004 Australia; 2 School of Population Health, University of Auckland, Auckland, New Zealand; 3 Victorian Spinal Cord Service, Austin Hospital, Melbourne, Australia; 4 Department of Civil, Environmental and Geomatic Engineering, University College of London, London, UK; 5 Trauma Service, The Royal Children’s Hospital, Melbourne, Australia; 6 Department of Paediatrics, University of Melbourne, Melbourne, Australia; 7 Surgical Research Group, Murdoch Childrens Research Institute, Melbourne, Australia; 8 Trauma Service, The Royal Melbourne Hospital, Melbourne, Australia; 9 Farr Institute at the Centre for Improvement in Population Health through E-records Research (CIPHER), Swansea University Medical School, Swansea University, Swansea, UK

**Keywords:** Trauma, Injury, Disability, Recovery, Communication, Health literacy, Interview

## Abstract

**Background:**

Navigating complex health care systems during the multiple phases of recovery following major trauma entails many challenges for injured patients. Patients’ experiences communicating with health professionals are of particular importance in this context. The aim of this study was to explore seriously injured patients’ perceptions of communication with and information provided by health professionals in their first 3-years following injury.

**Methods:**

A qualitative study designed was used, nested within a population-based longitudinal cohort study. Semi-structured telephone interviews were undertaken with 65 major trauma patients, aged 17 years and older at the time of injury, identified through purposive sampling from the Victorian State Trauma Registry. A detailed thematic analysis was undertaken using a framework approach.

**Results:**

Many seriously injured patients faced barriers to communication with health professionals in the hospital, rehabilitation and in the community settings. Key themes related to limited contact with health professionals, insufficient information provision, and challenges with information coordination. Communication difficulties were particularly apparent when many health professionals were involved in patient care, or when patients transitioned from hospital to rehabilitation or to the community. Difficulties in patient-health professional engagement compromised communication and exchange of information particularly at transitions of care, e.g., discharge from hospital. Conversely, positive attributes displayed by health professionals such as active discussion, clear language, listening and an empathetic manner, all facilitated effective communication. Most patients preferred communication consistent with patient-centred approaches, and the use of multiple modes to communicate information.

**Conclusions:**

The communication and information needs of seriously injured patients were inconsistently met over the course of their recovery continuum. To assist patients along their recovery trajectories, patient-centred communication approaches and considerations for environmental and patients’ health literacy are recommended. Additionally, assistance with information coordination and comprehensive multimodal information provision should be available for injured patients.

## Background

Recovery following major trauma can be protracted and complex involving repeated interactions with many health professionals over extended timeframes. Effective communication between patients and health professionals is highly important to build trust, share information, make collaborative decisions, foster adherence to prescribed medical treatments, and to reduce the risk of errors and harms [1, 2]. Effective communication from the patient’s perspective involves health professionals imparting clear information, inviting questions, actively listening to patients, not rushing interactions, and having a caring, respectful and empathetic manner [3]. Further, communication that ascertains patients’ needs, perspectives and values is also considered effective, as it a key feature of patient-centred care [4]. Issues with communication between patients and health professionals and among health professionals are evident in the literature across diverse clinical fields [5–10]. Harms associated with poor communication in health care include patient dissatisfaction and distress, negligence claims, poor quality of care, medical errors, adverse events, and sentinel events (resulting in serious patient harm) [11].

Many seriously injured patients experience multiple injuries, long recovery timeframes and persisting disability, resulting in interactions with numerous health professionals from different specialities as they transfer between hospital, rehabilitation and community settings. Previous studies have shown patients want detailed information about their injuries, recovery and health, and support services [12–14]. Patients also report receiving inadequate explanations of treatment options and being inconsistently engaged by health professionals in decision-making [15]. However, the experiences of seriously injured patients with health professionals and the factors affecting communication and information transfer, have not been explored in detail over an extended recovery timeframe. Therefore, in the first 3 years following injury, this study explored seriously injured patients’ perceptions of communication with and information provided by health professionals in hospital, rehabilitation and community settings.

## Methods

The Victorian health care system operates an integrated trauma system which coordinates prehospital and acute care across the state [16]. The Victorian State Trauma System ensures injured patients are delivered to the right hospital in the shortest time for definitive care and management [17]. Central to this system are two adult hospitals and one paediatric hospital designated as major trauma services (Level 1 trauma centre equivalent) [16].

This qualitative study, nested within a population-based longitudinal cohort study, was conducted in Victoria with major trauma survivors. Called the REcovery after Serious Trauma—Outcomes, Resource use and patient Experiences (RESTORE) project, the long-term outcomes and experiences of seriously injured patients were explored [18]. Patients with an injury date from 1 July 2011 to 30 June 2012 and registered with the Victorian State Trauma Registry (VSTR) were purposively sampled for interviews at 3-years post-injury. The VSTR inclusion criteria is defined as any of the following: death (at scene of injury or in-hospital) related to injury; admitted to intensive care for > 24 h; urgent surgery for intracranial, intrathoracic or intra-abdominal injury, or for fixation of pelvic or spinal fractures; or suffered multiple traumatic injuries (have an injury severity score (ISS) > 12) [16]. The ISS is an indicator of overall injury severity and has a value of zero to 75 [19].

To ensure a range of participants were represented, we sampled based on age, gender, compensation status, residential location (metropolitan or regional), and whether their care was delivered at a major trauma service or not. Patients were eligible for the study if aged 17 years and over when injured, and registered with the VSTR. The exclusion criteria were patients with a severe traumatic brain injury or spinal cord injury who have been studied separately as they have distinct information needs and care pathways, and non-English speaking patients, as use of interpreter services was logistically challenging for exploring in-depth perceptions of recovery. The Monash University Human Research Ethics Committee (CF14/915-2014000365) and participating hospitals approved the study.

At the conclusion of a structured 3-year post-injury follow-up interview, patients were invited to participate in an in-depth telephone interview [18]. When designing the study, experienced injury researchers estimated approximately 40–60 interviews would be required to meet the purposive sampling criteria and to ascertain the diverse experiences of seriously injured people. After 50 interviews, it was evident that participants were no longer reporting new information. An additional 15 interviews were obtained due to concerns about participant attrition, as the patients were also to be followed up at 4 and 5 years post-injury. Further, concerns about recall bias were discussed by the project team given the long period between hospitalisation, rehabilitation and the 3-year post-injury interview. However, 3 years after injury, it was expected that participants would focus only on the communication problems that had a major impact on their memory.

Interested patients were sent a participant information sheet and called 10 to 14 days later to address questions and schedule an interview time. Three trained qualitative interviewers (including the first author (SB)) performed the interviews using a topic guide developed from previous qualitative research in injured populations and informed by the research aims [15] (Table 1). This topic guide served to identify the main issues to be explored. Probes were used by the interviewers to further explore the topics in different settings and with different health professionals. The interviewers met regularly (at a minimum weekly) and reported to the broader project team weekly. At these meetings, the team discussed emerging themes, new information arising from the interviews, and whether to stop or continue interviewing. All interviews were conducted between July 2014 and July 2015, involved a median interview time of 47 min. Interviews were audio recorded and transcribed, and verbal consent to participate was recorded at the commencement of the interview.

**Table 1:** Interview questions

Interviewer prompts	
How do you feel about the care you received in hospital and rehabilitation? What was good and not so good about your care? What information or advice did you receive about your injury? Was this information written or verbal or both? Who provided you with information about your injuries? How useful was this information? Is there any other information you would have liked to have receive? Can you tell me about the types of treatments and services you are using now? How do you feel about the care you received with these services? Are these treatments and services meeting your needs? If not, how could they be improved?	

### Analysis

Interviews were loaded into NVivo 10 (QSR International, Doncaster) and a detailed thematic analysis was undertaken using a framework approach [20]. The framework approach was selected as it facilitated a systematic analysis of a large number of transcripts. This approach entails the development of a framework from interconnected stages of analysis. The framework represents the abstracted and conceptualised themes and subthemes, which when applied to the dataset, reveals the original links to the participants’ experiences [20]. To gain a comprehensive overview of the data, all 65 transcripts were read by the first author and a sample by multiple project investigators (also authors). The first author (SB) inductively analysed the data by initially coding selected passages in the transcripts based on content and meaning to identify recurrent themes and issues. Through an iterative process, patterns were analysed and labelled, and subsequently grouped into emerging themes. A framework of themes and subthemes was developed to reflect connections and discrete patterns. Two of the authors (SB and BG) developed the themes, and the project investigators who read a sample of transcripts, provided important input that shaped the analysis and framework. Consensus on the final themes was reached through discussion. Themes and subthemes were reviewed to construct an overall picture of the data.

## Results

Of the 65 adult major trauma patients interviewed, 82% (*n* = 53) received definitive treatment at a major trauma service. The median (range) time since injury was 3 (2.9–3.1) years. Most (74%, *n* = 38) were working for income prior to injury and 73% (*n* = 35) had returned to work at 3-years post-injury. Almost half (45%, *n* = 29) of the patients were funded by the Transport Accident Commission (TAC), the state’s ‘no fault’ insurer for road traffic injury, while 43% (*n* = 27) were funded by Medicare, Australia’s publically funded universal health care system. The remaining 12% (*n* = 8) were funded by private health insurance, WorkSafe (workers’ compensation), or their compensable status was unknown. The profile of the patients is shown in Table 2.

**Table 2:** Profile of patients (*n* = 65)

Descriptor			
Age	Mean (SD) *n* (%)	50.7 (15.5)	
	17–39 years	17 (26.1)	
	40–59 years	24 (36.9)	
	≥ 60 years	24 (36.9)	
Gender	*n* (%)		
	Male	42 (64.6)	
Self-reported pre-injury disability	*n* (%)		
	No	57 (87.7)	
Mechanism of injury	*n* (%)		
	Motor vehicle	22 (33.8)	
	Fall	12 (18.5)	
	Motorcycle	6 (9.2)	
	Pedal cyclist	6 (9.2)	
	Other^a^	19 (29.3)	
Injury severity score	Median (IQR)	17 (14–24)	
Length of hospital stay in days	Median (IQR)	11 (5.4–26.5)	
Discharge destination	*n* (%)		
	Home	38 (58.5)	
	Inpatient rehabilitation or hospital for convalescence	27 (41.5)	
36 month outcomes	GOS-E^b^	*n* (%)	
		Upper good recovery	19 (29.2)
		Lower good recovery	7 (10.8)
		Upper moderate disability	22 (33.9)
		Lower moderate or severe disability	17 (26.1)

^a^Other includes horse related, pedestrian, struck by/collision with object or person, unspecified external cause, other specified external cause, other threat to breathing, machinery, firearm, cutting piercing object or fire, flames or smoke ^b^Glasgow Outcomes Scale Extended (GOS-E), data has been collapsed from eight outcome categories

The 3-year outcomes for functional status was assessed using Glasgow Outcome Scale-Extended (GOS-E) and are shown in Table 2. This scale measured self-reported self-care, communication, cognition, relationships, activities of daily living, work and study, social and recreational participation [21] to categorise function on a scale from one (death) to eight (upper good recovery). The 36 month GOS-E outcomes revealed many patients with persisting moderate to severe disability.

Themes are presented with excerpts followed by a description of the patient’s gender, age group, broad cause of injury, injury type, phase of care and a unique identifying number. Excerpts have been drawn from all phases of care over the course of the patient’s recovery: hospital care; hospital discharge; rehabilitation care; rehabilitation discharge (if applicable); community care, to support the themes. Table 3 summarises the key recommendations arising from the results and patient recommendations.

**Table 3:** Key recommendations for communication improvement

Theme	Health professionals	Patients
Discharge planning	• Provide written information about post discharge services and points of contact for advice and assistance • Engage patients in collaborative discharge planning well before their expected date of discharge	• Initiate discussions with health professionals about discharge long before the expected date of discharge
Multimodal communication	• Provide information in different modes such as verbal, written text, pictures, and photographs	• Request written information and/or for information to be presented in alternative formats e.g. pictures, audio-visual etc.
Information provision and sharing	• Provide detailed explanations about patients’ injuries, treatments, expected recovery and future • Provide tailored information consistently and repeatedly throughout acute and recovery phases of care • Provide information in plain English to patients or in their preferred language • Ensure comprehensive and timely information is communicated to health professionals across care transitions involved in patient care	• Raise issues with health professionals during interactions, even if not asked • Ask health professionals to repeat information in plain language if the information is not clear or understood • Request to speak to a doctor privately if health professionals visiting in groups
Information coordination	• Check with patients how information provided fits with information received from other health professionals	• Request health professional assistance with integrating information from multiple health professionals if required • Have a trusted relative or advocate to assist with the coordination of information
Active communication	• Use communication approaches that are patient-centred • Ensure regular face-to-face contact with patients • Actively listen to patients and encourage them to share information and to ask questions • Respond to patient concerns with potential solutions	• Actively question health professionals during communication • Actively engage in communication with health professionals to obtain information about health, health care and services
Investigate	• Follow up on patients post discharge to check how they are managing	• Follow up on information that health professionals say will be organised, but does not eventuate • Follow up on unresolved issues in reasonable timeframes • Persist with finding health professionals that meet individual needs
Organisations		
	• Ensure information available to staff and patients with regards to contacting patient advocacy groups and when their services could be useful • Provide accessible translator services • Provide trauma coordinators to assist with cultural, information and services navigation • Ensure all notices, information and instructions, within the hospital and provided to patients, comply with best practice for health literacy	

## Information needs

### Information needs at phases of care

The provision of information by health professionals was highly valued by most patients. While some patients were satisfied with the information provided, the desire for more information was a consistent theme, albeit different information at various phases of care. The way in which this information was communicated was also of importance to patients.

#### Hospital care

In the hospital setting, while most patients acknowledged they received information, many wanted and expected more. Patients expressed the need for more information from doctors and nurses on why tests and procedures were required, test results, and prescribed medications and their side effects. Further, patients wanted personalised information about their injuries and severity, expected consequences of the injuries, possible need for surgery, likely recovery timeframes and expectations, and current and future plans of care. One patient outlined the specific information he would have liked to have received during his hospital stay:

“I would have liked more information at the early stages, exactly what was going on. Like a description of what my injuries were and what the treatment has been to that stage and why that treatment has been done, and what the future holds.” *Male_40–49yrs_non-transport injury_multiple injuries_hospital care_#211*

Of the information provided in hospital, many patients were satisfied with the way in which it was communicated. Doctors and nurses were often referred to as ‘reassuring’, ‘professional’, ‘clear’ and/or ‘respectful’ in their verbal communication. Some patients perceived specialist doctors’ communication as effective when information was freely provided about the extent of their injuries, treatments (including what was done in surgery), immediate plans, and prognosis. Detailed information was appreciated when it was communicated in different modes, such as verbal, written text, visual (e.g. pictures, drawings and photographs), as this improved patients’ understanding of complex and unfamiliar information, such as surgical procedures. At a time of uncertainty and confusion, one patient recounted a positive communication experience during her hospital stay:

“He (the surgeon) drew pictures for me. I knew exactly where the breaks were, where the plates were going to be inserted and how they were going to be inserted…. And he just explained it really, really well and the pictures were great.” *Female_40–49yrs_non-transport injury_head injury_hospital care_#228*

#### Rehabilitation care

Less than half the patients were discharged from hospital to rehabilitation. Those who experienced inpatient rehabilitation care were largely satisfied when doctors and nurses provided information on expected timeframes of care, details of long-term treatment options, the likely level of recovery, and the pros and cons of treatments. Patients were appreciative of information provided by nurses that identified the many health professionals responsible for their care, and that advised on how to stay positive during rehabilitation. One patient reported valuing the time taken by rehabilitation nurses to personally offer supportive communication:

“I’d say the nurses, the care, how personable they were towards you gets you through. There were a number of days where I’d given up, I’d had enough. And you just end up having a nurse in your room talking to you for 20 minutes, half-an-hour, just about whatever.” *Male_17–29yrs_road traffic injury_multiple injuries_rehabilitation care*_#*581*

Information provided by speech therapists, occupational therapists and physiotherapists was appraised favourably when it provided clear instructions on how improve and manage disability, regain or maintain strength, and use mobility assist devices. Many participants appreciated physiotherapists’ communicating recommended exercises, particularly when this information was physically demonstrated and personalised for the patient:

“The physio was good… he gave me exercises to do and just to strengthen up the areas that I damaged…he actually gave me instructions on what to do and showed me in which direction to move it.” *Male_50–59yrs_road traffic injury_multiple injuries_rehabilitation care_#169*

#### Inpatient discharge

Many patients described wanting more information prior to their discharge home. Information was sought from doctors and nurses about expected recovery levels and timeframes, plans for discharge, and the process and plan of care management after discharge. Information about follow up services and treatments in the community, recommended activities, scar and wound management, and who to contact for information and advice was also sought. Communication of this information was perceived to be generally poor as these information needs were often unmet. A number of participants suggested that information specifically about counselling/psychological services and the contact details, should have been provided at discharge:

“As I was leaving hospital, or before I was discharged, something could have been said about some kind of counselling or just some kind of number to contact.” *Female_30–39yrs_non-transport injury_multiple injuries_hospital discharge_#130*

As patients had primarily engaged with specialist doctors while in hospital (and rehabilitation, if attended), some sought clarification and details about the role of the general practitioner (GP) in their recovery after discharge. One patient who had never been hospitalised before reflected:

“It was never properly explained to me the role in my recovery of the (name of hospital) versus my local doctor. And it was only down the track that I discovered that it was my local doctor who had taken the handover in terms of… overseeing my recovery. That was never made clear to me.” *Male_17–29yrs_non-transport injury_multiple injuries_rehabilitation discharge_#101*

#### Community care

After discharge from hospital and/or rehabilitation, many patients were provided with outpatient appointments (public patients) or booked in to see their specialist/s (private patients). Some were also instructed to make a time to see a GP. However, in many cases these appointments were not scheduled for several weeks post-discharge. During this time (between discharge and the doctor’s appointment), patients reported needing information that related to medications, pain and wound management. Many patients expressed that they were uncertain about who was an appropriate health professional to obtain this information from:

“I came out of rehab on a very strong course of medication, and I really didn’t know who I should be speaking to about that… I wasn’t sure I needed it anymore but couldn’t get a definitive answer anywhere on that.” *Male_40–49yrs_road traffic injury_multiple injuries_community care_#611*

Up to 3 years post-injury, patients mainly interacted with surgeons, medical specialists (such as ophthalmologists or neurologists), GPs, psychologists, and physiotherapists. From surgeons and medical specialists, patients expressed the need for information on long-term treatment plans, recovery timeframes, managing ongoing disability, and pain management. Enduring disability of any level drove some patients to peruse information about how to improve their condition, as it often impacted negatively on their quality of life. One patient recounted that to obtain this information, it required a doctor who would acknowledge the personal impact of her disability and communicate in an empathic manner:

“I have lost taste and smell. When I did see the neurosurgeon, he said to me, ‘Well, get over it, get on with it’… For me, I find this very distressing... I’ve actually switched doctors because I want to know, is there anybody that can help me… I would like to be able to talk to somebody who could say is there any exercises that I would be able to train my brain in order to get those neurons working and the nerves working.” *Female_60–69yrs_non-transport injury_head injuries_community care_#992*

Patients consistently reported wanting GPs to provide information on managing, treating and reducing persistent physical and psychological disability and chronic pain, as well as return to work. Information on improving strength, fitness, range of motion in damaged joints, and increasing mobility was also desired from physiotherapists. While overall, patients were pleased with the information and treatment received from most physiotherapists, sometimes it took time to find one with a compatible communication style. Several patients reported similar difficulties with developing rapport and the communication of information when engaging with GPs and psychologists/psychiatrists:

“The guy that I spoke to was a psychiatrist, he couldn’t relate to me. He had done every qualification under the sun and he actually didn’t have any idea what I was talking about.” *Male_17–29yrs_non-transport injury_spinal and other injuries_community care_#266*

## Accessing, using and understanding information

### Clarity of information

Information delivered by health professionals using inaccessible language left many patients confused and dissatisfied. The use of medical terminology and words that exceeded the patient’s health literacy level in verbal and written information, impeded patients from developing an understanding of their condition and treatment. Some patients reported not understanding information delivered at the time of hospital discharge, and/or when communicating with surgeons in hospital:

“I suppose just a bit more of an overall understanding of what was (surgically) happening. So a bit more information, just of a general nature rather than specific medical sort of speak, just, I suppose in layman’s terms.” *Male_40–49yrs_non-transport injury_head injury_ hospital care_#568*

### Consistency of information

Inconsistent information from health professionals challenged injured patients’ abilities to understand their condition and treatment. Sometimes patients were provided with incorrect or conflicting information from health professionals through verbal communication and in written documentation. In this setting, the large number of health professionals involved in seriously injured patients’ care further exacerbated the issue. Inconsistent information typically related to hospital discharge times, differing opinions in the long-term surgical management of injuries, and/or the origins of symptoms. Confusion subsequently occurred regarding who or what information to believe. Sometimes inconsistency was evident in written information which left some patients unclear about how to move forward with their recovery:

“The discharge summaries, the one I got from (name of rehabilitation) and one I got from (name of hospital), are completely different in explaining what happened and what I can do now.” *Male_17–29yrs_road traffic injury_multiple injuries _community care_#860*

Another patient recalled how inconsistent information resulted in uncertainty about a diagnosis that had implications for different treatment pathways.

“My psychiatrist has got one opinion and the GP has another opinion, and they’re completely different opinions. So I don’t know if I go for the experience of the psychiatrist, or the GP, I don’t know. I’m not medically able to do that. I’m relying on them to tell me. I’d like to know if I got dementia or Alzheimer’s or it’s just symptoms of a closed head injury.” *Male_40–49yrs_road traffic injury_multiple injuries_community care_#689*

### Access to information

Some patients, who were not in contact with health professionals after hospital or rehabilitation discharge, felt isolated and unsupported in managing their injuries and emotional state at home. A lack of access to information meant some patients did not understand how to appropriately progress their recovery, such as whether to see a psychologist, how to find one, if and/or when to see allied health professionals, how to manage pain, and what exercises to do. A lack of knowledge to communicate with GPs or rehabilitation specialists at this stage placed patients at risk of further injury or delayed progress. Two patients recalled how they felt immediately after discharge:

“Because once you get your discharge it’s like you’re on your own. You got to do it yourself... you feel sort of alienated...” *Male_30–39yrs_road traffic injury_multiple injuries_community care_#688*

“… when I came out of hospital they were going to send me to a physio place where I stayed on the premises… but that didn’t come to fruition … and I found myself probably going to gym by myself or perhaps swimming by myself, and not sure what I was doing.” *Male_60–69yrs_non-transport injury_multiple injuries_ hospital discharge_#092*

## Information coordination

As the management of traumatic injuries typically involved specialists from a variety of units, such as orthopaedic, vascular, neurology and plastics, patients communicated with multiple health professionals while in hospital and at outpatient appointments. During these consultations, many patients reported receiving fragmented information about their injuries and the care delivered. Some patients reported discovering the full extent of their injuries only after hospital discharge. New information was revealed by rehabilitation and community health professionals, family members, legal documents, or hospital discharge summaries. Patients described the subsequent process of constructing a complete picture of their condition and injuries as challenging and time consuming. One patient recounted that she was unable to do this until after her discharge from rehabilitation:

“I didn’t have one particular person giving you all the information. It was just the medical staff as they came through. It was only at the end that I recall, that I got the information all put together.” *Female_60–69yrs_road traffic injury_multiple injuries_hospital & rehabilitation care*_#*415*

Family members played an important role in the coordination of information, particularly for the period of time patients reported being unable to process information themselves, which was typically in the hospital setting. Some of these family members were health professionals or had knowledge of health systems. Several patients mentioned the value of having a person to coordinate information on their behalf when in hospital:

“My wife, who is a division one nurse, was with me most of the time, and she would be passing on most of the information, and she’d be getting it off the doctors, because I was sort of half in a trance most of the time.” *Male_40–49yrs_non-transport injury_multiple injuries_hospital care_#335*

After hospital discharge, some patients expressed that having one person to coordinate information and serve as a single point of communication for patients and health professionals involved in their care, would have been beneficial:

“A case manager… someone that has a good look at everything and make sure that all the information is passed on to the patient, as well as anyone dealing with them: patient and family. It all seems to be like a big sort of a lot of people fixing different parts of you and no-one thinking to put all the information together and let you know, or anyone.” *Male_40–49yrs_road traffic injury_multiple injuries_community care_#773*

Another strategy to assist with the coordination of information proposed by multiple patients was the provision of written information. The impact of operative medications, analgesic side effects, emotional reactions to serious injuries and their suddenness, or mild to moderate head injuries, affected many patients’ abilities to comprehend and retain information. As verbal information was often relayed at times when patients’ cognitive function was suboptimal, many suggested that the provision of documentation could mitigate this issue:

“For me it would have been no good telling me anything at (hospital name). Perhaps if (hospital name) issued you ... a (written) summary of what your injuries were when you were brought in, what you were diagnosed with and resulting treatments that they performed.” *Male_17–29yrs_road traffic injury_multiple injuries_rehabilitation care_#581*

Some patients suggested that providing information later in the recovery process, when clarity had returned, would have enabled more informed and appropriate decision making:

“… having come off the medication, I had a lot more comprehension and ability to focus, and being taken through everything then, I might have done things a bit differently in my next steps.” *Male_40–49yrs_road traffic injury_multiple injuries_hospital care_#611*

Some patients expressed concern about the organisation of their information between hospital and primary care providers. The communication of information, such as follow-up appointments and hospital discharge summaries, was remarked by patients to be predominately the responsibility of health professionals. When insufficient, untimely or unclear information was transferred, some patients perceived their health to be unnecessarily compromised:

“I was told I was supposed to go back in a month’s time ... and have a follow up x-ray. When I rang to get that organised no-one knew about it (or) me and they had no idea what I was talking about… I didn’t have any more X-rays… but I still had broken ribs… So my right lung wasn’t working properly, and that’s why I got pneumonia.” *Male_40–49yrs_non-transport injury_multiple injuries_ community care_#533*

## Communication needs

### Favourable communication attributes

Patients stated favourable health professional communication attributes were active discussion, the use of simple clear language and the provision of rationales for planned courses of action, as these facilitated information exchange and understanding. Patients also valued frequent contact, a sensitive and attentive manner, personalising information, good listening skills, not rushing communication, and being responsive to their needs and questions. Such patient-centred communication was appreciated at all stages of recovery:

“Just the interest that they took in me and just the thoroughness of it all really. I could discuss it with lots of doctors. There was lots of people there I could talk to, it was always good.” *Female_50–59yrs_road traffic injury_multiple fractures_hospital care_#169*

“I could talk to him (GP) about anything. I started off seeing him twice a week, but if I needed to see him they always squeezed me in. I credit him a lot for my peace of mind. You can ask him anything… and he doesn’t treat you as if you’re stupid.” *Female_60–69yrs_non-transport injury_multiple injuries_community care_#*214

### Unfavourable communication attributes: a lack of patient engagement

In inpatient and outpatient settings some busy doctors did not actively engage patients in discussion. By not inviting questions, this lack of two-way communication obstructed the flow of information and issues of importance to patients were missed:

“I just think they (surgeons) could have asked me was there any issues, because I did have issues. I had a neck issue, and I still have a neck issue….” *Male_60–69yrs_road traffic injury_multiple injuries_community care_#381*

Many patients reported not being engaged in communication about discharge planning. Failure to consult patients early in the discharge planning process limited the amount of time available for the patient to address and resolve concerns. This was particularly challenging for the many injured patients who were in rehabilitation for extended periods and who were going to require ongoing service engagement (e.g. physiotherapy, occupational therapy, carers) when at home:

“So it seems like you’re going along, you’re doing your rehab, you’re attending, you’re making progress and then all of a sudden they’ll come to you and say okay, you’ll be finishing up in a couple of weeks – that’s it... it seems a lot like they don’t engage the patient very well.” *Male_17–29yrs_road traffic injury_multiple injuries_rehabilitation care_#102*

### Unfavourable communication attributes: dismissal of patient concerns

Sometimes health professionals were perceived to dismiss patient concerns, and/or fail to address patients’ problems. The inability to achieve shared understandings in communication left patients feeling frustrated and distressed. While this issue was reported to occur in all phases of care, in the community setting numerous patients conveyed that GPs, specialists or sometimes physiotherapists dismissed their concerns and/or failed to listen. In particular, persistent pain was a frequently reported problem. In some cases, patients sought the opinions of many different health professionals to get their needs heard. Despite their unmet needs, some patients felt powerless to challenge the authority of health professionals. One patient expressed her discontent when a GP failed to ascertain her perspective on pain management:

My GP, I’m not happy at all... all he does is write out narcotics (prescriptions). It’s more than one at a time. They are different ones, and to take together. I was asleep nearly all day and night. I can’t do that… He doesn’t even examine me… I feel as though I go in there and he just wants to get me out. *Female_60–69yrs_non-transport injury_multiple injuries_community care_#980*

## Discussion

The results of this study provide valuable insights into the communication and information needs of patients recovering from serious injury. Many seriously injured people found accessing, using and understanding injury and recovery information in the health care system to be challenging. Engaging with large numbers of health professionals from various specialities with different communication styles, resulted in variable communication effectiveness and quality of care for injured people. Difficulty coordinating information from different sources and unmet information needs persisted for many up to 3 years after injury.

Seriously injured people need quality health information to be effectively communicated to comprehend health issues, follow medical advice, and make appropriate decisions about treatment, recovery, and future plans [22, 23]. How health information is accessed, used and understood is affected by individual health literacy and the health literacy environment [22]. The health literacy environment is the health professionals, the policies and procedures of the service, and the infrastructure of the health system [22]. For seriously injured people in our study, the health literacy environment required them to navigate complex and unfamiliar language, deal with inconsistent and missing injury information, and integrate information provided from numerous and diverse health professionals over a prolonged timeframe.

Conversely, some health professionals communicated in a way that reduced the complexity of the health literacy environment, satisfying the information needs of injured patients. At all stages of the recovery pathway, patients perceived the communication of information to be favourable when multimodal communication methods (e.g., pictures, demonstrations) were used and information was presented in plain language. Similarly, in the context of orthopaedic surgery, Choi [24] developed and validated pictorial based discharge instructions for older adults after hip replacement surgery. Five health care experts reviewed the action-based discharge instructions, such as climbing stairs after surgery, and endorsed them as engaging and helpful for patients to follow. The communication style used by some health professionals in our study developed rapport with injured patients. Responsive and empathic communication by doctors was also found by Chu and Tseng [25] in a survey of orthopaedic patients having hip and knee replacements in two health centres in Taiwan. This application of patient-centred communication assisted people with low health literacy to comprehend pre-operative instructions. Patients in our study were also satisfied when health professionals communicated personalised information. For those with low health literacy, Wynia and Osborne [26] conducted a survey across 13 US health care organisations and found that poor health outcomes associated with low health literacy could be mitigated by health professionals using patient-centred communication styles.

Overall, these studies and the results of ours, suggest that reducing the health literacy demands on injured patients could better support access to and understanding of information. In a non-injury context, additional strategies to reduce health literacy barriers are documented in numerous local and international health literacy guidelines, tool kits and policies [27–30]. Further research is needed to understand how these strategies are best implemented in the context of serious injury.

Given poor health outcomes are associated with low health literacy [31], the application of universal precautions for health literacy are recommended [23]. These involves consistently communicating with patients in a way that reduces the complexity of health information, and supports information use and understanding without needing to measure individual health literacy [23]. In our study, while many patients reported effective communication, this was not consistently reported for any particular health profession, or at any stage of the recovery pathway. Indeed, information needs existed for severely injured people across all genders, ages, and disability levels. These findings highlight the need for improved health professional awareness and education on the importance of health literacy and how to reduce the complexity of health environments [32–37].

Individual health literacy is an important influence on active participation in health care. Individual health literacy is composed of the person’s skills, knowledge motivation and abilities to access, use and understand health care and information [22]. In our study, the health literacy skills of patients were affected by a lack of knowledge about their injuries and how the health system worked. For some, strong medication/s impeded clarity of thought and were a barrier to retaining information, a finding also identified by others [12]. Further, some patients lacked the advocacy skills and/or the confidence to communicate their own values and preferences to ensure quality health services were delivered. Patients, however, can be trained to actively communicate in a way that increases their ability to obtain information, participate in their health care, and receive person-centred care [38]. Such training could involve teaching patients to actively question health professionals, draw issues of concern to attention, ask for clarification, and to provide preferences during medical conversations [2, 39]. While such training is likely to benefit injured patients, important differences may not occur without concomitant changes in organisational policy and health professionals’ practices [22, 40].

Previous research into the health literacy skills and abilities of traumatically injured people is limited. Rosenbaum et al. conducted a survey of US patients presenting with foot and ankle injuries to an emergency department showed a high prevalence of low health literacy [41]. Shahan et al. also found trauma patients surveyed in a US outpatient department to have poor recall and knowledge of their injury and operations [42]. By focusing, however, on the measurement of patients’ abilities to read and recite, these results reflect a narrow approach taken by the researchers to measure health literacy. Our results expand understandings about health literacy for trauma patients by highlighting the challenges faced when communicating with health professionals, as well as in accessing, using and understanding health information over multiple phases of care and recovery.

Injured patients’ information needs persisted over time and changed as they recovered. Consistent with others who have studied injured patients [12, 15], many patients described wanting more information. The importance of meeting information needs is that preventable health issues can develop if barriers to communication and information exist [5, 7, 43]. Therefore, to promote understanding and information retention in the hospital setting, doctors and nurses need to provide personalised written and verbal information in plain language consistently and repeatedly throughout the patients stay. Specifically, patients wanted doctors to provide personalised information about current and proposed treatments plans for their condition at different stages of recovery, and to provide information on recovery timeframes and the expected level of recovery. Due to diverse levels of health literacy, assertiveness and communication competence in patients, health professionals should be responsible for initiating such conversations [44].

Discharge to home after a prolonged inpatient stay was described as stressful by many seriously injured patients, a finding consistent with others [12, 15]. Inadequate communication and information transfer at discharge can be a barrier to recovery [45], and a cause of non-compliance with medical care, unplanned re-admission, and preventable adverse events [46, 47]. To reduce anxiety and improve access to information about the expected date of, and plans for, discharge, health professionals need to work collaboratively with patients and family members [48]. Documentation of injuries, treatment plans and available services, would likely assist patients and their relatives with their efforts to coordinate information at discharge. To ensure patients understand and apply instructions about physical activity after discharge, doctors, nurses and physiotherapists can tailor information to a patient’s learning style and ask patients to ‘teach back’ the information [49]. Further, to ensure patients understand who to contact after discharge, the role of the GP or a rehabilitation specialist in advising, treating and linking the patient to other health professionals needs to be clarified. To facilitate comprehensive and timely communication and information sharing between health professionals as patients transition from the hospital/rehabilitation to community care [50, 51], electronic communication systems and follow-up phone calls from treating doctors to GPs could assist [50].

In the community, GPs were often the main point of contact and the pathway to accessing other specialists and health professionals. However, the quality of GPs and other services varied. Therefore, it is important that injured patients have some understandings or supports to assist them to navigate around GPs that fail to address their needs. Our results and those of Christie et al. suggest that written discharge plans from hospital (and rehabilitation, if applicable) could provide access to personalised information directly to patients about services, and how and when to connect to them, that could facilitate long-term recovery [14]. Additionally, a specialist trauma coordinator or trauma patient advocate dedicated to supporting patients and their family members could mitigate health literacy barriers to improve understandings about hospital and post-discharge care. By facilitating communication between health professionals, acting as a single point of contact, providing consistent, integrated and up-to-date information, and coordinating service provision, a trauma coordinator could reduce the complexity of the health system for injured patients [52, 53].

While the context of our study was seriously injured patients, it is likely our findings regarding their information and communication needs have relevance to other patients with complex health conditions and prolonged recovery trajectories. Further, the suggestions made to reduce the complexity of the health literacy environment, improve patients’ knowledge, support health literacy skill development and patient-centred communication, are issues of safety and quality of care, and therefore important to consider in other populations.

### Limitations

This study offers detailed insights into seriously injured patients’ diverse communication needs and experiences over a 3-year recovery continuum. Nevertheless, some limitations exist. This study was limited to reporting patient perceptions of communication only. It is possible health professionals may have a different perspective of communication with patients. Only individuals who could understand written and spoken English were included in the study. Therefore, the views of those with the added communication challenge of a language barrier are not represented. As the study involved exploring communication experiences over an extended period of time, it is possible that patients’ memories have faded or were at times affected by medication. However, patients freely declared in the interviews when they felt their memories were fallible. Additionally, many patients were still regularly interacting with health professionals, allowing them to draw on more recent experiences.

## Conclusion

The communication and information needs of seriously injured patients were inconsistently met over the course of their recovery continuum. The challenges to effective communication multiplied for seriously injured patients as during their recovery they encountered numerous health professionals, crossed over care settings, and often returned home with ongoing and complex needs. Recommendations for improvement require patient, health professional and organisational change. Organisations need to ensure the health environment is suitable for patients lacking health literacy skills and that the implementation of patient-centred communication approaches is supported. Health professionals need to actively engage patients, use multi-modal communication strategies and plain language to provide information repeatedly throughout the recovery process. Patients need to actively question health professionals, draw issues of concern to their attention, and ask for clarification when required. Many injured patients also require assistance with information coordination and integration over the course of their recovery.
